# Been there before? Examining “familiarity” as a moderator for discriminating between true and false intentions

**DOI:** 10.3389/fpsyg.2014.00677

**Published:** 2014-07-07

**Authors:** Melanie Knieps, Pär A. Granhag, Aldert Vrij

**Affiliations:** ^1^Psychology Department, University of GothenburgGothenburg, Sweden; ^2^Norwegian Police University CollegeOslo, Norway; ^3^Department of Psychology, University of PortsmouthPortsmouth, UK

**Keywords:** deception detection, episodic future thinking, familiarity, mental images, true and false intentions, prototypical prospection

## Abstract

Prospection is thinking about possible future states of the world. Commitment to perform a future action—commonly referred to as intention—is a specific type of prospection. This knowledge is relevant when trying to assess whether a stated intention is a lie or the truth. An important observation is that thinking of, and committing to, future actions often evoke vivid and detailed mental images. One factor that affects how specific a person experiences these simulations is location-familiarity. The purpose of this study was to examine to what extent location-familiarity moderates how liars and truth tellers describe a mental image in an investigative interview. Liars were instructed to plan a criminal act and truth tellers were instructed to plan a non-criminal act. Before they could carry out these acts, the participants were intercepted and interviewed about the mental images they may have had experienced in this planning phase. Truth tellers told the truth whereas liars used a cover story to mask their criminal intentions. As predicted, the results showed that the truth tellers reported a mental image significantly more often than the liars. If a mental image was reported, the content of the descriptions did not differ between liars and truth tellers. In a post interview questionnaire, the participants rated the vividness (i.e., content and clarity) of their mental images. The ratings revealed that the truth tellers had experienced their mental images more vividly during the planning phase than the liars. In conclusion, this study indicates that both prototypical and specific representations play a role in prospection. Although location-familiarity did not moderate how liars and truth tellers describe their mental images of the future, this study allows some interesting insights into human future thinking. How these findings can be helpful for distinguishing between true and false intentions will be discussed.

## Introduction

Prospection is thinking about possible future states of the world. This capacity allows people to better foresee, plan, and shape their futures (Suddendorf and Corballis, 2007). Commitment to perform a future action—commonly referred to as *intention*—is a specific type of future thinking. An important observation is that thinking of, and committing to, future actions often evoke vivid mental images (Goschke and Kuhl, 1993; Pham and Taylor, 1999). Also, mental images are more easily constructed if the future event is related to a personal goal (D'Argembeau and Mathy, 2011). Hence, being personally committed to a future event affects the construction of mental images.

Prospection, and mental images in particular, have recently also become of interest in deception research related to future actions (e.g., Granhag and Knieps, 2011; Warmelink et al., 2012; Knieps et al., 2013a,b). The goal of this research is to distinguish between individuals telling the truth about their intentions and individuals trying to mask their criminal intentions with a cover story (false intentions). In brief, an intention is defined as an actor's mental state preceding a corresponding action which is inherently accompanied by a commitment to perform a specific action. Intended acts are often preceded by some degree of reasoning and planning and are directed at the intender's own action (Malle et al., 2001). Being able to detect deception about future actions has clear societal value. At best, it could prevent crimes before they happen. The main assumption in this study is that true intentions correspond to the processes underlying prospection whereas false intentions may not correspond to the same processes. Hence, false intentions may be accompanied by less vivid mental images than true intentions; a difference which may be reflected in an investigative interview. Research on prospection may be helpful in capturing the possible differences between true and false intentions.

### Prospection and retrospection

People often engage into *mental time travel* to re-experience the past (i.e., retrospection) and pre-experience the future (i.e., prospection) (Tulving, 1985; Atance and O'Neill, 2001). Research shows that our prospective ability rests to a great extent on our retrospective ability. More precisely, our ability to imagine future events with detailed and vivid mental images relies on memory, and on episodic memory in particular (for reviews see, e.g., Szpunar, 2010; Schacter et al., 2012). In brief, scholars generally distinguish between *semantic memory* (i.e., the recall of general factual knowledge) and *episodic memory* (i.e., the memory of personally experienced events). For example, a person may well remember the last time he or she visited Rome, and having a particularly self-indulgent dinner at a famous restaurant in the city center. But if asked what is the capital of Italy this person may simply say “Rome” without experiencing any personal recollections of the many times he or she has been there. Memory for the dinner is episodic. Knowledge that Rome is the capital of Italy is semantic.

So far, research on prospection has mainly focused on the specific or “episodic” representations of the future emphasizing its subjective experience (such as visual perspective and emotion, D'Argembeau and Van der Linden, 2004; Van Boven and Ashworth, 2007). In the scientific literature this phenomenon is often referred to as *Episodic Future Thinking* (EFT; Atance and O'Neill, 2001; D'Argembeau, 2012). Besides these specific representations, recent research indicates that more prototypical or “semantic” representations play an important role in prospection as well (Kane et al., 2012; Martin-Ordas et al., 2012). *Prototype* refers to “the class of generalized mental representations of what people, events, and activities are typically like—their archetype, their typical attributes, the scripts by which they usually unfold, the instances that best exemplify an event, and so on” (p. 355, Kane et al., 2012). It is possible that mental images of the future contain both specific and prototypical pieces of information. This is an important finding as statements that containing specific contents, a feature associated with episodic memory, is regarded as an indicator for the truth in the psycho-legal literature (Steller and Köhnken, 1989). Hence, there is a risk that a truthful statement may mistakenly be taken for a false statement just because it lacks the specific contents associated with the truth.

One factor that moderates how vividly people experience mental images is familiarity. Research shows that people, when thinking about events in the near future, tend to imagine themselves in familiar contexts interacting with familiar people (D'Argembeau and Van der Linden, 2004; Hassabis and Maguire, 2007; Gamboz et al., 2010). Comparing the vividness of events shows that representations of unfamiliar contexts typically contain fewer sensorial details, occur in a less clear context, and have a weaker subjective experience than representations of familiar contexts (Szpunar and McDermott, 2008). Hence, the contents of simulations of the future are usually characterized by familiar, contextual information.

### True and false intentions

Recent research has examined whether mental images may be helpful in discriminating between true and false intentions (Granhag and Knieps, 2011; Knieps et al., 2013a,b). In these studies participants were asked in an investigative interview whether they had experienced a mental image while planning their future action. The results show that almost all truth tellers did in fact report a mental image whereas a participant who did not report a mental image was typically a liar. If a mental image was reported, truth tellers tended to provide relatively more words to describe it (Granhag and Knieps, 2011; Knieps et al., 2013b). However, the content of the description did not reveal differences between liars and truth tellers (Knieps et al., 2013a,b). However, when asked to rate the vividness of their mental image in a post interview questionnaire, truth tellers tended to experience their mental images more vividly than liars (Granhag and Knieps, 2011; Knieps et al., 2013b). However, this effect seems to be moderated by the temporal distance between experiencing a mental image in the planning phase and how it is perceived a week later (Knieps et al., 2013a).

All studies had in common that the participants planned to execute an action in a location they were most likely to be familiar with (i.e., the local shopping mall in town). As a consequence, liars may have benefited from being familiar with the location. Memory research suggests that familiarity influences how vividly we experience mental images of the future (Szpunar and McDermott, 2008). Research on true and false intentions should therefore examine how familiarity affects the statements of liars and truth tellers in an investigative interview.

### The present study

The purpose of the present study is to examine to what extent location-familiarity moderates how liars and truth tellers describe a mental image in an investigative interview. Our main assumption is that truth tellers provide richer descriptions of their mental images than liars. This assumption rests on the finding that imagining a self-relevant future event is accompanied by a stronger feeling of experiencing and more episodic details (deVito et al., 2012). We assume that cover stories are less self-relevant, if at all, than true intentions.

In brief, half of the participants (the *liars*) planned a mock criminal act (searching for an envelope containing illegal material to deliver to an unknown person in the library). They were also asked to plan a cover story, to be used to mask their criminal intentions if stopped and questioned. The remaining half of the participants (the *truth tellers*) was asked to plan a non-criminal act (choosing a course based on the literature to be found in the library). Hence, the future actions that the participants were supposed to perform had an explicit goal, and the external constraints (i.e., the where, when, and what to perform) were clearly defined. All participants were asked to rate their familiarity with respect to different libraries. Based on these ratings, they were assigned to a library they either had never been to before (unfamiliar-condition) or to a library they were highly familiar with (familiar-condition).

We tapped possible differences between liars and truth tellers in two different ways. First, we analyzed the liars' and the truth tellers' descriptions of their dominant mental image during an investigative interview. For truth tellers such a mental image was related to the truthful intentions stated, and for liars this mental image was related to the false intentions stated (the cover story). In particular, we examined to which extent the descriptions contained features associated with episodic memory. Second, we analyzed the liars' and the truth tellers' experiences of their mental images that they had rated in a post interview questionnaire. In particular, these ratings tried to capture the vividness comprising different measures on clarity and content of the mental image (see Arnold et al., 2011).

With reference to the EFT framework (e.g., Szpunar, 2010) and previous empirical findings (Granhag and Knieps, 2011; Knieps et al., 2013a,b), we predict that more truth tellers than liars will report that they had experienced a mental image when planning their stated future actions (Hypothesis 1). Furthermore, if a mental image was described during the interview, we predict that the truth tellers' descriptions will contain more details than the liars' (Hypothesis 2a). The type of details under investigation were (a) *target library* (a particular location), (b) *navigating in the library*, (c) *navigating to the library* (e.g., taking the tram to the location), (d) *visual details* (physical information, such as objects and people), (e) *perception/sensation* (e.g., sounds, emotions, stress), and (f) *obstacles* to achievement of the goal (e.g., time pressure, other people, books unavailable). We predict that truth tellers who were familiar with the location will provide more details in their descriptions than truth tellers who were unfamiliar with the location (Hypothesis 2b). With respect to the self-ratings of the post interview questionnaire, we predict that the truth tellers' mental images will be more vivid than the liars' mental images (Hypothesis 3a). Furthermore, we predict that truth tellers who were familiar with the location will experience their mental image more vividly than truth tellers who are unfamiliar with the location (Hypothesis 3b).

## Methods

### Participants

In total, 120 subjects (76 women, 44 men) between 19 and 58 years of age (*M* = 27.78, *SD* = 7.47) were recruited to participate in the experiment. They were guaranteed a compensation of an equal value of 100 SEK for their participation (approximate value of 15 USD). Additional monetary rewards were announced if participants were able to convince the interviewer of their veracity.

### Design

A 2 (Veracity: Truthful vs. Deceptive) × 2 (Location-Familiarity: Familiar vs. Unfamiliar) between-group factorial design was used. The liars were instructed to plan a criminal (mock) event (*n* = 62), and the truth tellers were instructed to plan a non-criminal event (*n* = 58). Based on their previous experience, half the participants, the *familiar participants*, planned their future actions in a university library they had visited before (*n* = 57) while the other half, the *unfamiliar participants*, planned their future actions in a university library they had never visited before (*n* = 63).

### Procedure

#### Screening

After being welcomed by the experimenter, all participants were asked to sign an informed consent form and answer some questions on how familiar they were with four local university libraries. We selected these libraries because of their proximity to the Psychology Department and because of the similarity of their interior layout (course literature and check-out desks on the entrance floor). First, the participants indicated whether they had ever been to the particular library before (“Yes, I have visited this library before” or “No, I have never visited this library before.” If participants answered “yes,” the next question asked them to rate their familiarity with this library on a 7-point scale (1 = *not familiar*, 7 = *highly familiar*). Subsequently, they were asked to rank their familiarity with each library. Only people who rated their familiarity with 5 or higher were considered for the familiar-condition. If participants had never visited the libraries, they were considered for the unfamiliar-condition. To achieve comparability we used approximately the same number of participants from the two conditions for each library. We also established a balanced gender distribution for the different conditions and the four libraries to eliminate them as potential confounders.

#### Planning phase

The participants were told that they had to successfully achieve a certain goal (criminal vs. non-criminal) within a certain time. They were informed that they were free to decide themselves exactly how they were about to achieve their goals. More precisely, the goal of the truth tellers was to “choose a course based on the course-literature to be found in the library” (the non-criminal event). By comparison, the goal of the liars was to deliver an envelope containing “illegal” material to an unknown person waiting close to the check-out of a particular library (the mock criminal event). A second task for the liars was to plan a cover story with the main theme “choose a course based on the course-literature to be found in the library” to mask their criminal intentions. This story was to be used if they were intercepted, and to achieve high comparability between the events that liars and truth tellers would later tell about in the investigative interview. That is, we provided the liars with a *frame* for their cover story (i.e., the *where, when*, and *what*) but made it clear that it was up to each participant to fill this frame in order to construct a convincing cover story (the *how*).

The participants had eight minutes time to plan and were offered more time (four more minutes) if needed. Only eight liars and five truth tellers asked for extra time. However, they had only one chance to plan the action. They were instructed to carefully plan their future actions and to finish all necessary planning before leaving the Psychology Department. They received a map of Gothenburg, the locations of all university libraries in Gothenburg, and time tables for public transport. Furthermore, all participants were given a photo of an individual: the truth tellers were told this was an individual who could assist them at the library if they needed any help finding the book(s) whereas the liars were told this was the individual to hand the envelope to at the library.

Also, all participants had access to a brochure of the courses taught at University of Gothenburg. Although participants were not explicitly instructed to use it, liars could use this brochure to prepare a better cover story and truth tellers could use it to facilitate choosing a particular course. In addition, the participants were motivated to plan by a number of explicit constraints; they were instructed that they had only one chance to carry out the task, and that they had a limited amount of time at their disposal. In short, our experiment used a set-up that encouraged the participants to imagine themselves acting at a particular time and place in the near future. After they had finished their planning, we determined that they had understood the instructions.

#### Interview

Immediately after the planning phase, the participants were brought to a nearby room where they expected to receive their tram tickets. Instead, they were given a list of instructions that asked them to imagine they would undergo a security check at the library entrance. However, when trying to pass this security check, they had been selected for further questioning. For this imaginary scenario, the liars were told to use their cover story, as convincingly as possible, to hide their criminal intention whereas the truth tellers were told to tell the truth, as convincingly as possible, about their true intention. That is, both groups were asked to speak about their book-search but while this story was mirroring the truth tellers' actual intention (true intentions), the book-search was just a cover story for the liars (false intentions). All participants were informed that the interviewer did not know whether they were lying or telling the truth and they were instructed to try to be as convincing as possible. Before leaving the room, the experimenter made sure that each participant understood the instructions. Then, all participants were interviewed individually with a structured interview protocol asking about their intentions, planning activities and the occurrence of a mental image during the planning phase. The interview was audio taped and the interviewer was blind to the participants' truth status.

#### Post interview questionnaire

Immediately after the interview, the participants were asked to complete self-ratings in a post interview questionnaire. The liars were explicitly instructed to cease role-playing and to answer this questionnaire truthfully. A manipulation check was conducted with the aim of controlling for some basic elements. Then the participants rated the degree of veracity of what she or he had stated in the interview on a 7-point scale (1 = *everything I told was true*, 7 = *everything I told was untrue*). In addition, the participants were asked to rate the following basic features of the planning phase on a 7-point scale: the difficulty of the planning phase (1 = *very easy*, 7 = *very difficult*); how sufficient they found the time allocated for the planning phase (1 = *not at all sufficient*, 7 = *totally sufficient*); how satisfied they were with the planning (1 = *not at all satisfied*, 7 = *very satisfied*); and how stimulating (interesting) they found the planning (1 = *not at all stimulating*, 7 = *very stimulating*).

Before completing the next section of the post interview questionnaire, the truth tellers were instructed to “Think back on the planning,” whereas the liars were instructed to “Think back on the planning of the cover story.” This section had ten questions related to EFT: two global dimensions and eight specific dimensions of the mental image. Participants rated these questions on 7-point scales (1 = *to a very low extent*, 7 = *to a very high extent*). The first global question was “To what extent did you form a mental image while planning your errand [cover story]?” This question was followed by a set of questions based on the *Memory Characteristics Questionnaire* (MCQ) that is based in the reality-monitoring framework (Johnson and Raye, 1981). Other researchers have used this questionnaire to map subjective perceptions when people pre-experience the future (e.g., see D'Argembeau and Van der Linden, 2004; Szpunar and McDermott, 2008).

The eight questions about the participants' subjective experiences of specific dimensions concerned the mental image they may have visualized in the planning phase. Three questions addressed sensorial details (the degree to which the mental image was characterized by (i) visual information, (ii) auditory information, and (iii) smell/taste information). Three questions addressed spatial information (the extent to which the mental image was characterized by clarity with respect to (i) the spatial location *per se*, (ii) the spatial location of objects, and (iii) the spatial location of persons). Two questions addressed temporal information (the extent to which the mental image was characterized by clarity with respect to (i) the time of the day and (ii) the temporal order of the event). We emphasize again that the liars were instructed to think about and rate their mental images when planning their cover stories (not the mental images when planning the criminal events). The last question was the second global question: “To sum up, how clearly did you pre-experience the future event?” (for the truth tellers), and “To sum up, how clearly did you pre-experience your cover story?” (for the liars).

#### Coding

All interviews were transcribed verbatim from the audio-tapes. The answers to the question “At any point during your planning, did you evoke a mental image of the future event?” were coded (“Yes, a mental image was evoked,” or “No, a mental image was not evoked.”). Two coders, who did not know who was a liar and who was a truth teller, read and re-read the answers carefully in order to assign them to one of six dimensions of the type of details. One coder rated all the answers (100%) and the other rated only some answers (44%). They used a 7-point scale (1 = *to a low degree*, 7 = *to a high degree*). The agreement between the coders measured by the Intra-class Correlation Coefficient (ICC) for consistency is considered acceptable/high. The ICC statistics on the six dimensions are as follows: (1) the *target library* (ICC = 0.910), (2) the *navigation in the library* (ICC = 0.887), (3) the *navigation to the library* (ICC = 0.782), (4) *visual details* (i.e., all physical information such as objects, people; ICC = 0.828), (5) *sensation/perception*, which addressed what they felt, sensed or how clearly they experienced their mental image (ICC = 0.752.), and (6) *obstacles* which may have kept them from achieving their goal (e.g., time pressure, people, not finding book; ICC = 0.710).

## Results

### Veracity

After the interview all participants rated (on a 7-point scale) the extent to which they had lied during the interview. Liars (*M* = 4.33, *SD* = 2.00) rated the degree of lying significantly higher than the truth tellers (*M* = 1.90, *SD* = 1.27), *t*_(104)_ = 8.14, *p* < 0.001, *d* = 1.486. This result confirms that the participants followed the instructions to lie or tell the truth.

### Familiarity

With respect to the familiarity with the experiment location (1 = *not familiar at all*, 7 = *highly familiar*), the participants in the familiar-condition were at the upper end of the scale ranging from 5 to 7 (*M* = 5.88, *SD* = 0.803). This result confirms these participants were highly familiar with the location and that our allocation was valid. However, we found no difference between the familiar liars (*M* = 5.83, *SD* = 0.805) and the familiar truth tellers (*M* = 5.93, *SD* = 0.813), *F*_(55)_ = −0.471, *p* = 0.639, η^2^ = −0.124.

### Planning

We conducted a 2 (Veracity: Truthful vs. Deceptive) × 2 (Location-Familiarity: Familiar vs. Unfamiliar) MANOVA with the four planning ratings as dependent variables (i.e., *satisfaction with planning, satisfaction with time, perceived difficulty of planning*, and *perceived stimulation of planning*). The Location-Familiarity main effect was not significant [*F*_(4, 111)_ = 2.061, *p* = 0.091, η^2^ = 0.069], but the Veracity main effect [*F*_(4, 111)_ = 13.985, *p* < 0.001, η^2^ = 0.335] and the Veracity × Location-Familiarity interaction effect were significant [*F*_(4, 111)_ = 2.910, *p* = 0.025, η^2^ = 0.095].

At the univariate level, the Veracity factor revealed significant effects for *satisfaction with planning, perceived difficulty of planning*, and *perceived stimulation of planning.* That is, the truth tellers (*M* = 5.43, *SD* = 1.40) were more satisfied with their planning than the liars (*M* = 4.61, *SD* = 1.55), *F*_(1, 115)_ = 9.158, *p* = 0.003, η^2^ = 0.074. Critically, both groups rated their satisfaction at the upper end of the scale. Furthermore, the liars (*M* = 3.85, *SD* = 1.64) perceived their planning as significantly more difficult than the truth tellers [*M* = 2.62, *SD* = 1.60; *F*_(1, 115)_ = 17.52, *p* < 0.001, η^2^ = 0.132]. Finally, the liars (*M* = 5.23, *SD* = 1.49) experienced the planning as significantly more stimulating than the truth tellers (*M* = 3.79, *SD* = 1.69), *F*_(1, 115)_ = 27.08, *p* < 0.001, η^2^ = 0.191.

Regarding the interaction effect, univariate tests revealed a significant effect for the *perceived stimulation of planning, F*_(1, 115)_ = 7.048, *p* = 0.009, η^2^ = 0.058. In other words, liars and truth tellers rated their planning as equally stimulating (or interesting) when they were unfamiliar with the location. However, when the location was familiar, truth tellers (vs. liars) perceived the planning as significantly less stimulating (see Table 1).

**Table 1:** **Means (and standard deviations) of planning for Veracity and Location-Familiarity**.

**Subjective ratings**	**Truth tellers**		**Liars**	
	**Familiar**	**Unfamiliar**	**Familiar**	**Unfamiliar**
Satisfaction, planning	5.63 (1.21)	5.20 (1.54)	4.71 (1.44)	4.52 (1.66)
Satisfaction, time	5.93 (1.84)	5.70 (1.77)	5.50 (1.45)	4.91 (1.74)
Planning, difficulty	2.22 (1.42)	2.97 (1.71)	3.82 (1.52)	3.88 (1.76)
Planning, stimulating	5.16 (1.40)	4.84 (1.63)	5.32 (1.42)	5.15 (1.56)

### Objective measures of the mental image (interview)

#### Reporting a mental image during the interview

A chi-square analysis revealed that significantly more truth tellers than liars reported a mental image (truth tellers: 54 of 58 (93.1%); liars: 43 of 62 (69.4%); χ^2^ (*N* = 120) = 11.18, *p* = 0.001). This result lends support to Hypothesis 1. No difference was found between the familiar participants (48 of 57; 84.2%) compared to the unfamiliar participants (49 of 63; 77.8%); χ^2^ (*N* = 120) = 0.799, *p* = 0.371).

#### Type of details

We conducted a 2 (Veracity: Truthful vs. Deceptive) × 2 (Location-Familiarity: Familiar vs. Unfamiliar) MANOVA (with the six self-ratings pertaining to the descriptions of the mental image as the dependent variables). We found a significant multivariate effect for Location-Familiarity [*F*_(6, 84)_ = 6.73, *p* < 0.001, η^2^ = 0.326]. However, the Veracity main effect [*F*_(6, 84)_ = 0.983, *p* = 0.442, η^2^ = 0.066] and the Veracity × Location-Familiarity interaction effect were not significant [*F*_(6, 84)_ = 0.981, *p* = 0.444 η^2^ = 0.065]. Thus, we failed to find support for Hypothesis 2a and Hypothesis 2b (see Table 2).

**Table 2:** **Means (and standard deviations) of the type of details for Veracity and Location-Familiarity**.

**Objective ratings**	**Truth tellers**		**Liars**	
	**Familiar**	**Unfamiliar**	**Familiar**	**Unfamiliar**
Library	4.69 (1.83)	2.46 (1.17)	4.59 (1.74)	3.48 (1.54)
Navigation in library	3.73 (1.64)	2.29 (1.36)	3.50 (1.92)	2.38 (1.28)
Navigation to library	1.69 (1.35)	2.46 (1.92)	2.00 (1.77)	2.67 (2.24)
Visual details	4.86 (1.36)	4.57 (1.43)	5.00 (1.51)	4.86 (1.28)
Perception/sensation	2.18 (0.91)	2.36 (1.25)	2.09 (1.34)	2.10 (1.41)
Obstacles	3.18 (1.47)	3.50 (1.12)	2.59 (1.26)	3.67 (1.46)

Note that all ratings for the liars pertain to how they perceived their cover story.

Univariate tests for Location-Familiarity revealed significant main effects for *target library*, [Familiar: *M* = 4.65, *SD* = 1.77; Unfamiliar: *M* = 2.9, *SD* = 1.42; *F*_(1, 93)_ = 26.891, *p* < 0.001, η^2^ = 0.224] and for *navigating in the library*, [Familiar: *M* = 3.63, *SD* = 1.76; Unfamiliar: *M* = 2.33, *SD* = 1.31; *F*_(1, 93)_=16.085, *p* < 0.001, η^2^ = 0.147]. Unfamiliar participants (*M* = 2.55, *SD* = 2.04) spoke more about *navigating to the library* than the familiar participants (*M* = 1.83, *SD* = 1.55). This difference bordered on significance [*F*_(1, 93)_ = 3.699, *p* = 0.058, η^2^ = 0.038]. Thus, individuals who were familiar with the library referred to this location and navigating *in* this location to a significantly greater extent than individuals who were unfamiliar with the library. In comparison, the participants who were unfamiliar with the library tended to refer to navigating *to* the library more often than participants who were familiar with the library. Unfamiliar participants (*M* = 3.57, *SD* = 1.26) addressed *obstacles* to a significantly greater degree in their mental images than familiar participants [*M* = 2.89, *SD* = 1.39; *F*_(1, 89)_ = 6.434, *p* = 0.013, η^2^ = 0.067]. Familiar (*M* = 2.14, *SD* = 1.13) and unfamiliar participants (*M* = 2.24, *SD* = 1.32) did not differ with respect to *perception/sensation; F*_(1, 89)_ = 0.120, *p* = 0.730, η^2^ = 0.001) and *visual details* [Familiar: *M* = 4.93, *SD* = 1.42; Unfamiliar: *M* = 4.69, *SD* = 1.36; *F*_(1, 89)_ = 0.556, *p* = 0.458, η^2^ = 0.006]. In other words, familiar and unfamiliar participants referred to visual details and their perception and sensation to a similar degree.

### Subjective measures of the mental image (post interview questionnaire)

We conducted a 2 (Veracity: Truthful vs. Deceptive) × 2 (Location: Familiar vs. Unfamiliar) MANOVA with the eight subjective ratings of the specific dimensions pertaining to the participants' perception of their mental image as dependent variables. The two global ratings were excluded from the MANOVA as they constitute combined ratings. We found significant multivariate effects for Veracity [*F*_(8, 106)_ = 3.917, *p* < 0.001, η^2^ = 0.228] and Location-Familiarity [*F*_(8, 106)_ = 3.641, *p* = 0.001, η^2^ = 0.207], but no Veracity × Location-Familiarity interaction effect [*F*_(8, 106)_ = 0.828, *p* = 0.580, η^2^ = 0.059].

#### Vividness of the specific dimensions

Regarding Veracity, the analysis of each dependent variable showed three significant main effects. First, the sensory dimension revealed differences for the *visual information* [truth tellers: *M* = 6.09, *SD* = 1.26; liars: *M* = 5.48, *SD* = 1.51; *F*_(1, 115)_ = 5.831, *p* = 0.017, η^2^ = 0.048], and *smell/taste* [truth tellers: *M* = 1.74, *SD* = 1.25; liars: *M* = 1.34, *SD* = 0.77; *F*_(1, 115)_ = 4.66, *p* = 0.033, η^2^ = 0.039]. Hence, truth tellers experienced visual information and smell/taste to a greater extent than liars. It is noteworthy that the visual information was (unlike smell/taste) rated at the higher end of the scale, which indicates that this aspect plays an important role in the visualization of future events. Unexpectedly, the liars perceived the *position of other people* significantly more clearly than the truth tellers [truth tellers: *M* = 3.12, *SD* = 1.79; liars: *M* = 4.23, *SD* = 1.85; *F*_(1, 114)_ = 10.54, *p* = 0.002, η^2^ = 0.085]. Hence, Hypothesis 3a was partially supported.

Regarding the Location-Familiarity, the analysis of each dependent variable showed significant main effects for three ratings: *visual information* [Familiar: *M* = 6.16, *SD* = 1.16; Unfamiliar: *M* = 5.43, *SD* = 1.55; *F*_(1, 115)_ = 8.36, *p* = 0.005, η^2^ = 0.068], *location* [Familiar: *M* = 5.77, *SD* = 1.16; Unfamiliar: *M* = 4.65, *SD* = 1.69; *F*_(1, 115)_ = 17.32, *p* < 0.001, η^2^ = 0.131], and *position of objects* [Familiar: *M* = 5.63, *SD* = 1.34; Unfamiliar: *M* = 4.52, *SD* = 1.79; *F*_(1, 115)_ = 13.82, *p* < 0.001, η^2^ = 0.107]. Thus, the participants who were familiar with the library experienced the location itself and how objects were arranged more clearly than the participants who had never been in the library before. Furthermore, the familiar participants perceived the visual information of their mental images more clearly than the unfamiliar participants.

#### Vividness of the global dimensions

In addition, we conducted two 2 (Veracity: Truthful vs. Deceptive) × 2 (Location-Familiarity: Familiar vs. Unfamiliar) ANOVAs on the two subjective ratings of the global dimensions pertaining to (a) the extent to which participants visualized mental images in the planning phase and (b) the overall clarity of the mental image. The results revealed that the truth tellers (*M* = 5.52, *SD* = 1.19) experienced their mental image of the future significantly more clearly than the liars [*M* = 5.00, *SD* = 1.44; *F*_(1, 115)_ = 4.67, *p* = 0.033, η^2^ = 0.039]. However, no significant differences were found for Location-Familiarity [*F*_(1, 115)_ = 3.15, *p* = 0.079, η^2^ = 0.027] or for the interaction effect [*F*_(1, 115)_ = 0.91, *p* = 0.343, η^2^ = 0.008] (see Table 3).

**Table 3:** **Means (and standard deviations) of the mental images' vividness for Veracity and Location-Familiarity**.

**Subjective ratings**	**Truth tellers**		**Liars**	
	**Familiar**	**Unfamiliar**	**Familiar**	**Unfamiliar**
**GLOBAL**				
Extent	5.54 (1.50)	5.33 (0.96)	5.46 (1.64)	4.88 (1.67)
Overall clarity	5.60 (1.1)	5.09 (1.35)	5.56 (1.22)	5.15 (1.26)
**SPECIFIC**				
Visual	6.54 (0.92)	5.76 (1.33)	5.79 (1.26)	5.16 (1.67)
Auditory	1.89 (1.47)	1.97 (0.98)	1.93 (1.27)	2.38 (1.50)
Smell/taste	2.00 (1.44)	1.52 (1.02)	1.32 (0.67)	1.38 (0.87)
Location	6.07 (0.90)	4.79 (1.37)	5.46 (1.32)	4.72 (1.82)
Position, objects	5.57 (1.33)	4.62 (1.55)	5.54 (1.37)	4.41 (1.98)
Position, people	3.36 (1.79)	2.90 (1.61)	4.14 (1.86)	4.41 (1.79)
Time of day	3.54 (2.03)	3.48 (2.18)	2.89 (1.75)	3.53 (1.95)
Temporal order	4.75 (1.80)	5.03 (1.57)	4.75 (1.74)	4.84 (1.54)

Note that all ratings for the liars pertain to how they perceived their cover story.

Regarding the extent to which participants visualized mental images in planning their future actions, the results revealed no main effect for Veracity [*F*_(1, 115)_ = 0.942, *p* = 0.334, η^2^ = 0.008], no main effect for Location-Familiarity [*F*_(1, 115)_ = 2.11, *p* = 0.149, η^2^ = 0.018], and no interaction effect [*F*_(1, 115)_ = 0.500, *p* = 0.481, η^2^ = 0.004].

## Discussion

The main purpose was to examine to what extent location-familiarity moderates how liars and truth tellers describe a mental image in an investigative interview. Similar to a recent study by Warmelink et al. (2012), familiarity was not found to have a moderating effect in this respect. Yet, some important observations could be made with respect to the role of prototypical and specific representations in prospection. As suggested by the self-ratings, truth tellers tend to experience their mental images more vividly during planning than liars. Hence, truth tellers experience the future in a comparatively more specific manner. However, the descriptions of the mental images provided in the interview did not show the same difference between liars and truth tellers. In other words, the findings of this study show a discrepancy between mental images *described* (data derived from the interview transcripts) and mental images *experienced* (data derived from the post interview questionnaire) which suggests that both specific and prototypical representations play a role in prospection. How this observation can be helpful for distinguishing between true and false intentions will be discussed.

### Interview

#### Reporting a mental image

In line with our hypothesis we found that the truth tellers (93.2%) reported a mental image more often in an interview than liars (69.4%). We can think of two possible and complementing reasons for this finding. First, it is possible that true intentions were accompanied by more vivid mental images than false intentions. This explanation would support the assumption that prospection, and evoking mental images in particular, is affected by the commitment of a person to execute a specific future action. Second, it is possible that the liars and the truth tellers used different strategies for handling the interview situation. Liars typically tend to keep their stories simple and try to avoid revealing possibly incriminating information in an interview whereas truth tellers tend toward “telling it like it was” and “keeping the story real” (Granhag and Strömwall, 2002; Strömwall et al., 2006). Hence, it is possible that the liars did not report a mental image in order to avoid that this piece of information could be used against them.

In conclusion, the different strategies, in combination with a less distinct mental image, may explain why fewer liars reported a mental image than truth tellers. This finding which successfully replicated past research (Granhag and Knieps, 2011; Knieps et al., 2013a,b) suggests that if a person does not report a mental image, it is likely that this person is a liar.

#### Content of the mental image

No differences were found with respect to the content of the mental images liars and truth tellers described during the investigative interview. This is noteworthy considering that the self-ratings indicate that such a difference exists between liars and truth tellers when they plan their future actions. One explanation for this finding is that truth tellers were not able to properly verbalize the vividness they experienced during planning when asked to describe their mental images in the investigative interview. As a consequence, the descriptions of liars and truth tellers were indistinguishable.

Our direct comparison between familiar and unfamiliar participants showed that participants have the tendency to talk about familiar themes in an investigative interview. More precisely, the familiar participants spoke about familiar themes related to the setting (the target library and navigating in the library) whereas the unfamiliar participants spoke about familiar themes different from the setting (navigating to the library). In other words, both familiar and unfamiliar participants relied to a large extent on prototypical information. In line with previous research (Bar, 2007; Kane et al., 2012), this observation indicates the relevance of prototypical representations in prospection. Furthermore, we argue that prototypical representations can, at least in part, explain why liars' and truth tellers' descriptions of their mental images provided during the investigative interview did not reveal any differences.

In conclusion, it is possible that both liars and truth tellers relied on more prototypical information to describe their mental image in the interview, irrespective of whether they were able to provide more specific information. In fact, liars and truth tellers mainly referred to spatial information in their descriptions which suggests that they may have used information from an existing prototype of a library.

#### Familiarity

Another finding from the direct comparison between familiar and unfamiliar participants was that unfamiliar participants addressed obstacles more often than familiar participants. Foreseeing potential obstacles is associated with more extensive planning (Xiao et al., 1997; Mumford et al., 2001). To date, the exact relation between specific representations of the future such as EFT and planning is still rather unclear (Szpunar, 2010). However, recent research indicates that planning requires both specific and prototypical representations (Martin-Ordas et al., 2012). The results of the present study suggest that the necessity to plan may affect to which extent we deliberate about obstacles. Differently put, it is possible that the unfamiliar participants were more motivated to plan, and to foresee obstacles for that matter, because they had never been to the particular library.

### Post interview questionnaire

#### Vividness of the mental image

As predicted, the truth tellers experienced the extent to which they visualized the future while planning, and specifically the sensory information, more vividly than the liars. In contrast to the prediction, the liars experienced the position of other people significantly more clearly than the truth tellers which may be due to that liars attributed more relevance to other people than truth tellers. All participants knew there would be a person in the library who was part of the study. However, it was optional for truth tellers to approach this person (to get help if needed); liars were required to approach this person (the envelope was supposed to be delivered to this person). This finding indicates that the relevance that a detail has for accomplishing a personal goal affects how clearly we experience this detail. This observation adds support to the link made earlier between specific representations of the future and goal-achievement (D'Argembeau and Mathy, 2011).

Regarding location-familiarity, the results suggest that spatial information plays a fundamental role in prospection. More precisely, the familiar participants experienced the *location* and the *position of objects* more clearly than the unfamiliar participants. This lends support to the view that the process of *scene construction* is critically involved in future thinking (Hassabis and Maguire, 2007). Scene construction entails retrieving and integrating perceptual, semantic, and contextual information into a coherent spatial context. However, although generic aspects such as those relating to the location are certainly fundamental elements for the construction of a more concrete mental image of the future, scene construction alone may lack the episodic flavor associated with EFT.

#### Planning

The results show that the planning of the future actions was more difficult for liars than for truth tellers. This may also explain why the liars were less satisfied with their planning. These two findings are not surprising as describing events in detail is typically more cognitively challenging for liars than for truth tellers (Vrij, 2008). It can be argued that this difference was caused by that liars had more to plan than truth tellers. However, that liars and truth tellers both show a strong satisfaction with the time allocated for planning works against this explanation. The lack of a difference between familiar and unfamiliar participants indicates that the planning did not cause problems for the participants who had never been to the particular library before. Hence, being unfamiliar with the location was not more challenging than being familiar with it, but lying was experienced as more challenging than truth telling. This finding indicates that increasing the cognitive load further may have an effect on liars in particular.

## Limitations

The concept of familiarity has some limitations. First, as we measured familiarity by the participants' self-ratings, our categorization depends on the accuracy of those ratings. However, we do not see any reason to doubt the reliability of the ratings. Second, as we only compared participants with rich memory of the location with participants with no memory of the location, there are many levels of familiarity between these two extreme positions that we did not consider in the present study.

Our research reflects future events in which the external conditions (i.e., the *where, when*, and *what*) are clearly defined. However, this is not the case for all statements about future events that require veracity assessments. Hence, the generalizability of our findings is therefore limited. Although this research covers only some of the possible future-related scenarios, there are numerous scenarios in real life (e.g., airport security checks) where most external conditions are specified at the time of questioning.

In this study liars were required to plan two tasks (a criminal action and a cover story) whereas truth tellers were required to plan only one task (a non-criminal action). In order to exclude the possibility that this difference between the conditions would result in a confound, we offered both liars and truth tellers more time to plan if needed. However, only 13 out of 120 participants (eight liars and five truth tellers) took advantage of this extra time. Furthermore, we employed self-ratings which revealed that both liars and truth tellers were satisfied to a high extent with the time allocated for planning. These findings suggest that both liars and truth tellers had enough time to plan their future actions.

## Future research

The results of this study indicate that although the self-ratings showed a difference between how vividly liars and truth tellers experience their mental images, no such difference was captured by the interview. This finding may be due to too insensitive interview questions. That is, it is possible that people describe prototypical information when undergoing an investigative interview although they may have more specific information available. The truth tellers may have provided more specific information in their descriptions, but—without prompting—they did not think about all the details of their mental image. Asking people to describe the full mental image that they have experienced might be viewed as a true challenge. Warmelink et al. (2012) argue that the experienced information needs to be actively retrieved before it can be described. Without such prompting, there is a risk that people will not think (and tell) about the details they experienced. This is supported by research which shows that people often have a considerable amount of information available that they do not convey without prompts (Fisher, 2010; Fisher et al., 2011). A recent study which examined the effect of memory-enhancing techniques lends support to the assumption that the framing of the questions help truth tellers (but not liars) to actively retrieve details from episodic memory (Sooniste et al., submitted).

The use of insensitive questions may also explain why location-familiarity was not found to moderate liars' and truth tellers' descriptions during the investigative interview. The role that specific and prototypical representations play in prospection and how this knowledge can be used to frame questions that more efficiently address genuine EFT is a topic for future research.

## Conclusions and psycho-legal implications

The results of the present study provide important insight into how people think of, and commit to, a future action. Lie-catchers may benefit from these findings on prospection and we will make some suggestions on how to use this knowledge in the investigative process. First, we believe that unanticipated questions should be asked. Questioning the participants about a mental image they may have “seen” while planning the future actions resulted in a greater amount of liars who denied having experienced such an image. This result suggests that if a person does *not* report a mental image in an interview, then he or she is very likely to be a liar. Second, be very careful in categorizing a statement as truthful just because it contains a great deal spatial details. A popular instrument to assess the veracity of written statements in several West-European countries, such as Sweden, Germany and the Netherlands and in parts of the USA, the Criteria-Based Content Analysis (CBCA) (Vrij, 2008), regards the quantity of detail in a statement as an indicator for the truth. Our study indicates that people tend to rely on prototypical information, and spatial information referring to locations and navigating in particular, when describing a mental image of the future. Hence, there is a risk that a false statement may mistakenly be taken for a true statement just because it contains a detailed description of the location. Lie-catchers should rather focus on more specific representations of the future such as EFT which emphasizes the subjective experience of prospection (such as visual perspective and emotion, D'Argembeau and Van der Linden, 2004). Third, identify the elements that are important and necessary for an individual to execute the stated intention such as the obstacles the planner may face when trying to execute the planned actions. Even though we found no differences between liars and truth tellers with respect to obstacles in this study, it appears to be of relevance in prospection. It may be worthwhile in future research to examine whether suspects address these aspects in their descriptions of a mental image. Lying is a cognitively demanding task (Vrij, 2008) and answering may be more difficult for individuals who have not genuinely planned to execute their stated intention.

In conclusion, the results of this study lend partial support to the assumption that specific representations of the future such as EFT are helpful in discriminating between true and false intentions. Although no clear differences were found in the content of liars' and truth tellers' descriptions of their mental images, it may be too hasty to reject the assumption that EFT is a helpful concept for eliciting cues to deception. The absence of differences between liars and truth tellers may be due to methods that were too insensitive to elicit descriptions reflecting genuine EFT. Framing questions that more efficiently address genuine EFT is a topic for future research.

### Conflict of interest statement

The authors declare that the research was conducted in the absence of any commercial or financial relationships that could be construed as a potential conflict of interest.
